# Dynamics, gene transfer, and ecological function of intracellular and extracellular DNA in environmental microbiome

**DOI:** 10.1002/imt2.34

**Published:** 2022-06-20

**Authors:** Mao Ye, Zhongyun Zhang, Mingming Sun, Yu Shi

**Affiliations:** ^1^ Key Laboratory of Soil Environment and Pollution Remediation, Institute of Soil Science Chinese Academy of Sciences Nanjing China; ^2^ University of Chinese Academy of Sciences Beijing China; ^3^ Soil Ecology Lab, College of Resources and Environmental Sciences Nanjing Agricultural University Nanjing China; ^4^ State Key Laboratory of Crop Stress Adaptation and Improvement, School of Life Sciences Henan University Kaifeng China

**Keywords:** ecological function, extracellular DNA, functional genes, intracellular DNA, metagenomics

## Abstract

Extracellular DNA (eDNA) and intracellular DNA (iDNA) extensively exist in both terrestrial and aquatic environment systems and have been found to play a significant role in the nutrient cycling and genetic information transmission between the environment and microorganisms. As inert DNA sequences, eDNA is able to present stability in the environment from the ribosome enzyme lysis, therein acting as the historical genetic information archive of the microbiome. As a consequence, both eDNA and iDNA can shed light on the functional gene variety and the corresponding microbial activity. In addition, eDNA is a ubiquitous composition of the cell membrane, which exerts a great impact on the resistance of outer stress from environmental pollutants, such as heavy metals, antibiotics, pesticides, and so on. This study focuses on the environmental dynamics and the ecological functions of the eDNA and iDNA from the perspectives of environmental behavior, genetic information transmission, resistance to the environmental contaminants, and so on. By reviewing the status quo and the future vista of the e/iDNAs research, this article sheds light on exploring the ecological functioning of the e/iDNAs in the environmental microbiome.

## Highlights

- Extracellular or intracellular DNA (e/iDNA) extensively exists in soil environment.
- e/iDNA indicates the functional gene variety and microbial activity.
- e/iDNA can transform into each other biologically.
- e/iDNA is essential for nutrient transformation and gene communication.

## Graphical abstract

Extracellular DNA or intracellular DNA (e/iDNA) extensively exists in the soil environment. e/iDNA indicates the functional gene variety and microbial activity. e/iDNA can transform into each other biologically. e/iDNA is essential for nutrient transformation and gene communication.

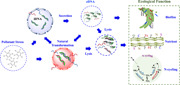

## INTRODUCTION

Best known as the general carrier of the genetic information of organisms, DNA is also a component of environmental organic matter, which can be further divided into extracellular DNA (eDNAs) and intracellular DNA (iDNAs) [1–3]. eDNA is cell‐free, naked DNA that usually persists in both aquatic and terrestrial environments as a polymer; the DNA that exists in the living cell is the so‐called iDNA [4, 5]. Mainly being released by living cells or the lying of dead cells, eDNA extensively presents in soil, aquifer, and sediment environments, and involves in environmental processes including absorption, degradation, natural transformation, and gene transmission [6–8]. Meanwhile, as an important component of bacterial biofilm, eDNAs are actively involved in the primarily substrate adsorption and bacteria aggregation processes for the construction of biofilm, thereby resisting the toxic antibiotics, heavy metals, organic pollutants, and so forth [9, 10]. In contrast, iDNAs mainly take part in the metabolic activity and lateral and horizontal gene transfer within cells. One of the main sources of eDNAs is the release of iDNAs by cells in the environment [11, 12]. This study mainly focuses on the distribution and the environmental behavior of eDNAs/iDNAs in the environmental microbiome (e.g., soil, seawater, and sediment), the role of eDNAs in the biofilm formation of type strains, the effect of eDNAs/iDNAs in the gene transmission, thereby reviewing the difference between two DNA fractions in the environmental behavior and the genetic information harbored. This study sheds light on the difference and interaction in ecological functioning between eDNAs and iDNAs, therein stimulating the further exploration of the corresponding mechanism between the two DNA fractions.

## ENVIRONMENTAL DYNAMICS OF EDNA/IDNA

**Figure 1:**
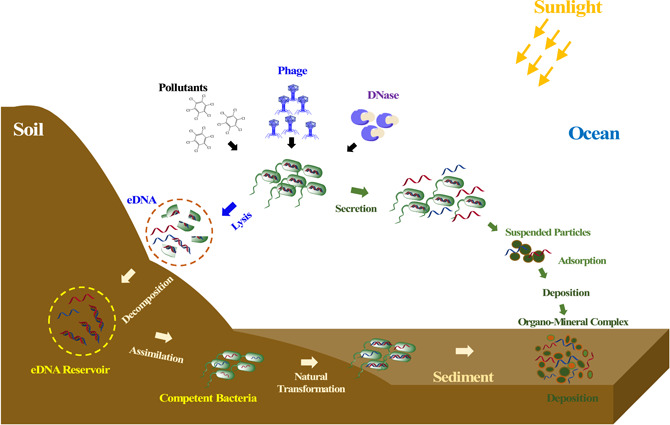
The environmental behavior of extracellular or intracellular DNA (eDNA/iDNA) in soil, seawater, and sediment. Processes including autophagocytosis, virus infection, active transport, and so on are the main pathways of eDNAs production. eDNA extensively presents in soil, aquifer, and sediment environments, and involves in the environmental processes including absorption, degradation, natural transformation, and so on.

As concluded in Figure 1, both living and dead cells can produce eDNAs through dead cell lysis or living cell exudate [12, 13]. Therefore, processes including autophagocytosis, virus infection, active transport, and so forth are the main pathways of eDNAs production [11, 14, 15]. Marine sediment is the largest DNA reservoir in the ocean environment, the amount of which is 10 times that of the living organisms in the ocean. It was estimated that there are approximately 0.45 × 10^9^ t eDNAs in the marine sediment, which account for 70% of the overall DNA in living cells [7, 16]. Environmental eDNAs can be classified into three groups, including free‐living, loosely bound, and tightly bound eDNAs fractions [6, 17]. It has been reported that living bacteria can either use the free‐living eDNAs as a nutrient source to support metabolism or incorporate the free‐living eDNAs into the chromosome [13, 18–21]. The loosely and tightly bound eDNAs are usually combined with organic matter through an inorganic cation bridge of phosphate groups or divalent cations, therein resisting the nuclease degradation, and existing persistently in the environment [8, 22, 23]. However, it is important to note that there is no distinct barrier among the three DNAs fractions. When environment factors vary, for example, pHs, temperature, and mineral features, two bounded eDNAs fractions might be desorbed from the organic matter, and degraded to participate in the matter cycling processes [13, 24, 25]. In contrast to eDNAs, iDNAs exist within living cells, thus avoiding direct interaction with environmental organic matters. Consequently, the environmental behavior and ecological functions of iDNAs differ from those of eDNAs. As described in Figure 1, iDNAs mainly involve nutrient cycling and metabolism, and lateral and horizontal gene transfer [26–28]. Meanwhile, the transformation of iDNAs into eDNAs happens when the iDNAs are released by living cells passionately or actively to maintain bacterial metabolism or counteract environmental stress [29]. Considering the distinguished roles between eDNAs and iDNAs in ecological service, it is important to develop a methodology that can separate eDNAs from iDNAs precisely, therein estimating the respective account of eDNAs/iDNAs in microbial communities, and determining the corresponding contribution of both DNAs in matter cycling and gene transformation in the environment. Conventional extraction methods containing filtration, centrifugation, and alcohol precipitation, could only retain about 10% of the total eDNA, while other frequently used methodologies, such as cetyltrimethylammonium bromide (CTAB)‐based extraction, and DNA extraction kits were more suitable for sediments, sludge, biofilm, and eDNA‐abundant soil samples. For aquatic samples, the DNA concentrations are too low for eDNA extraction, but magnetic beads were alternatively used to extract eDNA [3, 6, 8].

### Environmental abundance

We reviewed the abundance of eDNAs and iDNAs in various environmental systems. As concluded in Table 1, the content of both DNAs varied significantly among different environmental systems; soil, marine sediment, and seawater are the most important reservoirs of eDNAs and iDNAs. The eDNAs contents in soil, marine sediment, and seawater are in the range of 2.2–41.1 μg g^−1^, 0.03–119.1 μg g^−1^, and 5.1 ± 0.6 mg L^−1^, respectively, while iDNAs contents varies more significantly, between 0.1 ± 0.0 and 20843.3 ± 4077.6 μg L^−1^. It's important to note that eDNAs and iDNAs ubiquitously existed in different environmental systems, such as soil, sediment, seawater, sludge, manure, and so forth. eDNAs were generally more frequently detected than iDNAs; the abundance of eDNAs in soil and sediment were generally higher than iDNA, while the opposite trend was observed in aquifer systems, where higher iDNAs content was observed than eDNAs [8, 32]. Meanwhile, eDNAs content was usually higher in soil/sediment than that in aquifer [8, 41, 51].

**Table 1:** Abundance of extracellular (eDNA) and intracellular DNA (iDNA) in different environments

Environment	Environment	eDNA	iDNA	References
Water (ng ml^−1^)	Influent water	9.0–12.5	NA	[30]
	Effluent water	4.3–8.6	NA	[30]
	Waste water treatment plant	2.5 ± 0.4–15.9 ± 0.4	1.4 ± 0.2–10255.0 ± 162.6	[31]
	Waste water treatment plant	1.1 ± 0.0–7.8 ± 2.8	0.1 ± 0.0–20843.3 ± 4077.6	[31]
	Seawater	5.1 ± 0.6 × 10^3^	NA	[32]
	Seawater	1–88	NA	[33]
	Forest lake water	9–11	NA	[34]
	Drinking water	0.033–0.39	NA	[35]
Sediment (μg g^−1^)	Activated sludge	5.6–12.3 ng ml^−1^	NA	[30]
	Sludge	10.5–119.1	335.4–2047.4	[36]
	Sediment	0.94–6.23	2.78–17.13	[37]
	Livestock waste management structures sediment	0.6–9.2	190.0–1005.3	[38]
	Activated sludge	4.2–52.2 × 10^3^	NA	[39]
	Swine manure	8.6–9.7	179.0–295.4	[36]
	Sediment	0.023 ± 0.001	0.0005 ± 0.0001	[40]
	Aquatic sediment	6.7–24.3	0.31–0.67	[41]
	Aquatic sediment	77–116.8	63.7–89.7	[8]
	Sediment	2.3–57.7	NA	[29]
	Surface sediments	9.4 ± 3.0–22.5 ± 4.8	NA	[42]
	Sediment	0.0047 ± 0.0003–5.58 ± 1.26	NA	[24]
	Marine sediment	0.03–4.45 μg cm^−3^	0.03–5.84 μg cm^−3^	[43]
	Sea sediments	1.460–1.69	NA	[32]
	Marine sediment	3.3 ± 0.9–21.2 ± 1.3	NA	[24]
	Sea sediment	0.63 ± 1.59	0.035 ± 0.004	[40]
	Sea sediment	0.056 ± 0.001	0.010 ± 0.001	[40]
	Deep‐sea sediment	0.31 ± 0.18 g cm^−2^	NA	[16]
	Urban lake sediment	5.6–11.4	24.9–46.2	[36]
	Forest lake sediment	0.069–0.52	NA	[44]
	Ferruginous sediment	0.3–0.6	0.3–0.9	[45]
Soil (μg g^−1^)	Forest soil	2.2–41.1	NA	[46]
	Forest soil	7–55	29–197	[47]
	Forest soil	6.07	11.46	[48]
	Forest soil	1.70–1.90	NA	[49]
	Forest soil	6.0	NA	[50]
	Soil	0.08–1.95	NA	[44]

Abbreviation: NA, not available.

In the marine environment, it was estimated that the content of eDNAs in sediment was 3–4 times higher than that in seawater, which is likely caused by the strong adsorption of eDNAs to sediment organic matter, therein resulting in the longer half‐life period in sediment than that in seawater [24, 41, 52]. Meanwhile, the content of iDNAs is generally higher than that of eDNAs in the same seawater. DNA also extensively exists in surface soil. The percentage of eDNAs varies greatly from 0% to 83% according to previous studies [7, 53], which is likely caused by the extracting method and the varies in the soil properties [54].

### Environmental distribution and behavior

Upon release in the extracellular environment, molecular structure and environmental properties largely determined the distribution of eDNAs and iDNAs in soil, sediment, and seawater. The behavior of e/iDNAs in various environments has focused on the origin, adsorption kinetics, degradation pathways, gene transfer, etc. Being coated by a cell membrane, iDNAs behavior was closely correlated with predation by animals and microorganisms, and the transformation of iDNAs into eDNAs.

#### Origin

The accumulation of eDNA might derive from the lysis of dead cells, active or passive release by living cells following viral attack, grazing, exposure to cytotoxic agents [12, 55]. The excretion of intact cells from decomposing cells might depend on the activity and location of intracellular nucleases and reactive chemicals, while in dead cells, the degradation of iDNA to eDNA might be a slow process, relying on the conditions of these nucleases [12, 51, 55]. In most cases, the release of eDNA from bacterial and plant origins were considered.

Many genera of bacteria, such as *Acinetobacter*, *Pseudomonas*, *Bacillus*, *Micrococcus*, *Staphylococcus* could secret plasmid and chromosomal eDNA during their active growth or exposure to radiation and antibiotics [51, 56, 57]. It was considered that actively secreted eDNA was purer than that from dead cells, for the latter one was a mixture of inorganic salts, proteins, RNA, cell membrane residues, and other constituents [51, 57]. Moreover, the release of eDNA from plants varied a lot, which could take place after autolysis and decomposition of below‐ground biomass, mechanical disruption, and enzymatic degradation of cell structures by plant pathogens [12, 51]. During root growth, for example, the early corn root elongation, up to 10^3^ cells per primary root have been sloughed off per day, therein the majority of DNA was cleaved into small fragments by nucleases [12]. Mechanical disruption of plants membranes and cell walls led to the release of eDNA, but the abundant surrounding nucleases might degrade these eDNA after cell death. In addition, enzymatic activity of endoglucanases, polygalacturonases, and pectin methylesterases was enhanced by pathogen colonization, leading to the degradation of plant structures to release eDNA [12, 51]. The release of intact plant DNA by infecting bacteria might be important because of the potential accessibility of colonizing bacteria to other microbes [56, 57].

#### Adsorption

After release into the environment, eDNAs either exist as a free‐living state or are adsorbed by environmental minerals and organic matters [13, 58]. It was estimated that more than 80% DNA in marine sediment were eDNAs, among which more than 95% have been adsorbed by sediment substances [13, 16, 29]. Similarly, eDNAs and iDNAs can also be adsorbed by the abundant active functional groups at the soil particle surface. The adsorption of eDNAs to the soil particle protects it from degradation by nuclease by decreasing the response probability between eDNAs and the nuclease [59–62]. However, it was reported that eDNAs could still be partially degraded under the circumstances of nuclease supersaturation or the eDNAs and nuclease being adsorbed by the same mineral [61]. Therefore, soil properties including mineral composition, pHs, and humus and cation content exerted a cross‐impact on the adsorption kinetic of eDNAs in soil [63, 64]. Among various minerals, the adsorption capability of free‐living salmon sperm DNA was in the order of gabbro> montmorillonite> sericite; and the addition of Al^3+^ increased the DNA adsorption most significantly in comparison to Na^+^ and Mg^2+^ [65]. Similarly, Franchi et al. [66] also demonstrated that cation type clearly affected the DNA adsorption; cations with higher valence (Mg^2+^ and Al^3+^) stimulated the DNA adsorption by clay minerals in relative to Na^+^. In addition, Cai et al. [67] found that pHs also influenced the eDNAs adsorption to the organic clay and montmorillonite; eDNAs adsorption generally decreased with the pHs increasing from 2.0 to 5.0, which was barely detected at pHs higher than 5.0. Considering the difference between pure minerals and the minerals in the soil, studies were carried out to investigate the eDNAs adsorption to various minerals and mineral‐soil mixture [68]. It was found that the eDNAs adsorption by minerals in soil mixture declined by 1 to 2 magnitudes in contrast to the pure minerals [61, 64, 68]; but the adsorption content was still high as 200 mg g^−1^ soil.

In the marine environment, more than half of soluble eDNAs combined with particles, and became colloids in the seawater, as eDNAs can be adsorbed by the organic‐inorganic fine particle surface [58, 69, 70]. Currently, most researches focused on the role of marine environmental factors, including inorganic cation type and strength, water temperature, and water pHs, in the eDNAs adsorption in the seawater. Similar to the soil environment, cation types (Ca^2+^, Mg^2+^, and Na^+^) significantly impacted the adsorption of eDNAs in seawater; Ca^2+^ enhanced the adsorption of eDNAs to the organic matter, followed by Mg^2+^, while Na^+^ was favorable for the desorption of eDNAs from organic matter [71]. When it comes to the eDNAs in marine sediment, Lorenz et al. [72] found that the sandy particles in the marine sediments protected the adsorbed calf thymus eDNAs from desorption or nuclease degradation. Aardema et al. [52] investigated the impact of nuclease on the transformation of free‐living and adsorbing Bacillus subtilis eDNAs, and found that 200 ng ml^−1^ nuclease addition to the sediment resulted in the complete degradation of free‐living eDNAs, while adsorbed eDNAs maintained its transformation capacity even at the concentration of 8000 ng ml^−1^. Therefore, sediment particles significantly impeded the degradation of eDNAs by nuclease in the marine environment. In Xue et al. [73] study, the role of sediment and eDNAs type on the adsorption and desorption of eDNAs in seawater and marine sediment was investigated by real‐time PCR analysis. It was demonstrated that higher amount of eDNAs was adsorbed by sediment with more abundant organic matter or clay minerals. Meanwhile, the adsorption capacity of eDNAs in seawater was significantly higher than that in CaCl_2_ and MgCl_2_ solution, suggesting that cation type also impacted the adsorption of eDNAs in the aquifer system.

#### Degradation

Hydrolysis, oxidation, chemical cross‐linking, and exogenic nuclease degradation are all important processes that involve the degradation of eDNAs in the environment [74, 75]. The nuclease degradation of eDNA in soil, marine sediment, and seawater could be counteracted by the particle adsorption of eDNAs or nuclease.

In the soil environment, DNA structure and soil properties both determined the degradation of eDNAs, among which soil properties commonly played a more significant role than DNA structure [45, 51]. Sirois et al. [76] applied synthetic eDNAs to the soil, and investigated the impact of soil types, moisture, temperature, and agricultural management measures on the degradation dynamics of synthetic eDNAs; it was found that eDNAs quickly degraded in soils, which rate was positively correlated with soil moisture and temperature, while negatively associated with soil organic matter; and higher degradation efficiency was observed in fallow soil than that in tillage soil. Meanwhile, temperature increase significantly shortened the half‐life period of eDNAs in soil leachate, suggesting that degradation of eDNAs also belonged to enzyme reaction [77]. However, DNA could still be degraded slowly at temperatures lower than 0°C, indicating that eDNAs depletion in soil was the combined results caused by enzyme reaction, chemical hydrolysis, oxidation, and DNA cross‐linking [78]. Moreover, the content of soil contamination, such as arsenic, might have a more significant effect on iDNA degradation than eDNA. Take β‐glucosidase as an example which hydrolyzes nonreducing sugars, under the stress of 400 mg kg^−1^ As(V), the intracellular and extracellular β‐glucosidase activities decreased by 79% and 28%, respectively [79]. Other biotic or abiotic factors, for example, rapid desiccation, low temperatures, high salt concentrations, low pH, and a high content of clay minerals all might slow down the degradation of eDNA [17].

In seawater, Paul et al. [80] used ^3^H‐labeled thymidine to explore the production and transformation of *Escherichia coli* eDNAs; cellular nuclease and extracellular nuclease both degraded eDNAs, with the transformation period of dissolved eDNAs at 6.5 h. Borin et al. [81] found that high salinity lake water was favorable for the preservation of plasmid eDNAs for as long as 32 days. Meanwhile, it is interesting to note that pollutant presence in aquifers might also affect the degradation of eDNAs. For instance, Kang et al. [82] found that phenanthrene significantly inhibited the degradation of eDNAs in aquifer systems; and the higher content the of phenanthrene, the stronger the inhibition effect. This was likely caused by the fact that phenanthrene changed the eDNA structure and main chain composition, which resulted in the resistance of eDNAs to degradation.

Degradation of eDNAs in marine sediment also depends on the existence of nuclease. Dell'Anno et al. [32] found that the degradation rate of eDNAs was 7–100 times higher than that in marine sediment than that in seawater, which was mainly caused by the higher amount of nuclease in sediment. Meanwhile, Torti et al. [43] collected sediment from Aarhus Bugt at various depths and found that the proportion of small‐fraction eDNAs generally increased along with the increase in sediment depth, suggesting the increased degradation of large‐fraction eDNAs in sediment. It was believed that even at deep sediment, DNA nuclease still extensively existed, and stimulated the eDNAs degradation as a result. Upon binding of eDNA to humic substance, proteins, the accessibility of eDNAs to nucleases was decreased in marine sediments. Compared to dissolved fractions, the adsorption of eDNA to sand was more refractory, which required 100–1000 times more DNase and the adsorption of eDNA onto montmorillonitic clay required 10‐fold higher DNase to achieve the same extent of degradation, respectively [24, 55].

#### Natural transformation

**Figure 2:**
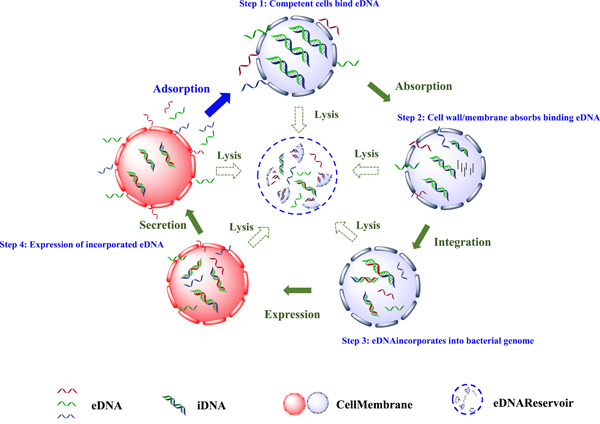
Pathways for bacterial natural transformation. Four critical pathways were included in natural transformation, (i) competent cells bind extracellular DNA (eDNAs); (ii) cell wall/membrane absorbs binding eDNAs; (iii) eDNAs incorporates into the bacterial genome; (iv) expression of incorporated eDNAs.

As a crucial behavior in the environment, the natural transformation of eDNAs is the main mechanism of eukaryotic absorption, incorporation, and expression of eDNAs. Natural transformation also called genetic transformation, is the process that which a competent cell absorbed free‐living DNA in the environment, and incorporates the absorbed DNA into its own genome. No specific protein is required for natural transformation, which makes the process happens fairly frequently in the natural environment. Four critical pathways were included in natural transformation, (i) competent cells bind eDNAs; (ii) cell wall/membrane absorbs binding eDNAs; (iii) eDNAs incorporate into the bacterial genome; (iv) expression of incorporated eDNAs [12] (Figure 2). The incorporated eDNAs, therefore, enter the gene transfer network through gene conjugation and transduction between bacteria [83]. The eDNAs that were not incorporated by the competent cell would be degraded quickly and joined the DNA metabolism cycling [12].

It is important to note that not all the e/iDNAs can be successfully incorporated into the bacterial genome, which was mainly dependent on the existence of a homologous region between the exogenous DNA chain and chromosomal DNA. It was reported that eDNA fraction at the length of 20–200 bp was suitable for the incorporation and further expression [83]. Vries et al. [84] found that the integration of eDNAs with low sequence homology into the genome of *Acinetobacter* sp. BD413 during transformation was at least 10^9^‐fold lower than that of homologous eDNAs. However, integration of eDNAs increased more than 10^5^‐fold when the eDNAs were linked to a piece of DNA homologous to the recipient genome.

The natural transformation of eDNAs is an important gene transfer mechanism in soil. Through homologous recombination, the competent bacterium *Acinetobacter* sp. BD413 acquired the eDNAs that encoded kanamycin resistance gene *nptII*, and showed kanamycin resistance characteristics afterward [85, 86]. Despite that most soil bacteria showed kanamycin resistance, the environmental risk of potential integration of kanamycin resistance gene by pathogenic bacteria through homologous recombination in soil deserves attention. Meanwhile, Khanna et al. [64] reported that the eDNAs state in the soil influenced the natural transformation; the adsorption of eDNAs to soil minerals decreased the frequency of natural transformation by one order of magnitude. Nielsen et al. [87] found that the natural transformation frequency varied significantly between eDNAs and *Acinetobacter* sp. BD413 in clay and sandy soils, which was three magnitudes higher in sandy soil than that in clay soil. It was concluded that the natural frequency rate of natural transformation was relatively higher in the hotspot, such as rhizosphere, bacterial biofilm, nutrient flow areas, and so forth than in nonhotspots in soil [1, 88].

Marine sediment and seawater are also important environments where natural transformation proceeds. As reported by Stewart et al., the frequency of natural transformation between eDNAs and *Pseudomonas stutzeri* in marine sediment was 4–20‐fold higher than that in water; sediment with higher content of organic matter showed a higher frequency of natural transformation than that with relative lower organic matter content [89]. In another similar study, however, no clear difference in the transformation frequency between broad‐host plasmid eDNAs and recipient Vibrio strain between marine sediment and seawater, both of which were at the range of 1.7 × 10^−6^–2.7 × 10^−10^ [90]. Therefore, factors including the transposons and nutrients applied could vary the frequency of natural transformation between eDNAs and recipient bacteria.

Currently, a total of 82 species have been shown to integrate eDNAs through a natural transformation in various environments, but the number may be an overestimate, lacking enough report documents and molecular proof [91, 92]. Once integrated by recipient bacteria, the genetic information carried out eDNAs can be further transferred to the environmental microbial community through conjugation and transduction. Therefore, more research is needed to investigate the role of eDNAs in matter cycling and ecological functioning as available transformative genetic information [51].

## ROLE OF E/IDNAs IN BIOFILM FORMATION

Biofilm has been ubiquitously detected in both terrestrial and marine environments to protect bacteria from adverse environmental disturbances, such as pollutant stressing [93]. Bacterial biofilm is a mixture of microorganisms, organic compounds, and inorganic substances; eDNAs, along with polysaccharides, fatty acids, proteins, and nucleic acids are organic components of biofilm [94, 95]. The bacteria biofilm has been found to be a cohesive three‐dimension network with high bacteria density, which is a favorable microenvironment for gene communication between bacteria through quorum sensing and horizontal gene transfer [96, 97].

Initial bacterial adhesion and surface aggregation are two critical steps that eDNAs involve in the formation of biofilm [98]. As an important component of the biofilm matrix, eDNAs not only introduce favorable acid‐base interaction but also provide the available thermodynamic condition for bacteria aggregation and adhesion to the surface [20, 99]. *Pseudomonas* (e.g., *Pseudomonas aeruginosa*) and *Staphylococcus* (*Staphylococcus aureus* and *Staphylococcus epidermidis*) are two kinds of genera that are commonly used to investigate the role of eDNAs in the biofilm formation. In this study, the biofilm formation of two genera in an aquifer environment is reviewed.

### P. aeruginosa

*P. aeruginosa* PAO1 is an opportunistic pathogenic bacterium with low pathogenicity, and strong drug resistance, which extensively exists in various natural environments. eDNAs have been shown to function as a structural support to maintain *P. aeruginosa* biofilm architecture [100].

The role of quorum sensing in the biofilm formation has been the focus of recent research due to the fact that it regulated the release of eDNAs vesicles from *P. aeruginosa* [101, 102]. It was found that Pseudomonas quinolone signaling (PQS) could induce the release of eDNAs from plankton. Meanwhile, Whitchurch et al. [28] demonstrated that eDNAs were an important component of biofilm as the application of DNase could inhibit the biofilm formation of PAO1. When applied DNase to the biofilm at different intervals found that biofilm at 84 h was more resistant to the DNase digestion than that at 12, 36, and 60 h, suggesting that biofilm could protect eDNAs from environmental disturbance.

**Figure 3:**
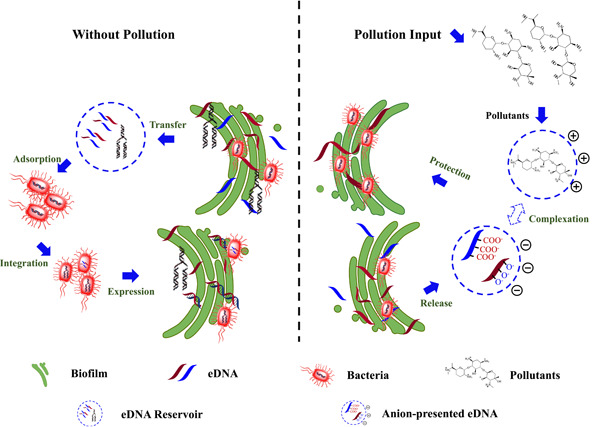
Mechanism of biofilm protecting bacteria from pollution. Extracellular DNA (eDNAs) can protect bacteria from the impact of pollutants by chelating cations (aminoglycosides, antibacterial peptide) in the biofilms. Moreover, eDNAs could be integrated into the biofilm of bacteria, which increased the resistance to pollutants.

In addition, eDNAs can protect PAO1 from the impact of pollutants by chelating cations (aminoglycosides, antibacterial peptides) and inducing antibiotic resistance in the biofilms [100, 103] (Figure 3). Chiang et al. [104] found that even exogenic eDNAs could be integrated into the biofilm of PAO1, which increased the resistance to aminoglycosides. Compared to the wild‐type *P. aeruginosa*, a DNA release‐deficient *P. aeruginosa* mutant was more susceptible to aminoglycosides but was rescued from the detrimental action of the drug upon supplementation with exogenous eDNAs.

### Staphylococcus

*Staphylococcus* has become serious nosocomial pathogenic bacteria that frequently cause infections [105]. Biofilm formation is considered a major factor determining its pathogenicity, which can be formed in both *S. aureus* and *S. epidermidis* to shield against antibiotics and autolysin [105–107]. Previous research has demonstrated the functions of eDNAs as important staphylococcal biofilm matrix polymers to initiate biofilm development. Moreover, the application of DNase I to the growth medium detached biofilms of *S. aureus* but not *S. epidermidis*, suggesting that eDNAs played different structural roles in *S. aureus* and *S. epidermidis* biofilms [106]. eDNAs in *S. epidermidis* biofilm were generated through the activity of the autolysin *AtlE* [105]. Qin et al. [107] demonstrated that eDNAs in *S. epidermidis* populations were mediated through *AtlE* lysis of a subpopulation of the bacterium, and the eDNAs promoted biofilm formation of the remaining population.

During biofilm formation of *Staphylococcus*, eDNAs regulated the genetic exchange between populations because of the multiple roles they played in the process, such as fundamental structure components, bacteria aggregation stimulator, surface adhesion regulator, nitrogen/phosphorus nutrients provider, and so on. The important role of eDNAs in biofilm pathogenicity was demonstrated in a previous study [107]. An isogenic *altE* mutant of *S. epidermidis* generated fewer eDNAs in the biofilm than that in the wild‐type bacterium, which resulted in the reduced infection capacity. In addition, the chelation between eDNAs and divalent cation in *S. epidermidis* biofilm increased the resistance of the biofilm to antibiotics. For instance, Natalya et al. [108] reported that eDNAs impeded the transport of vancomycin in *S. epidermidis* biofilms that were pre‐exposed to subinhibitory concentrations of vancomycin., suggesting that eDNAs protected the bacteria population in the biofilm from the antibiotic attack.

## BIOLOGICAL FUNCTION AND GENE TRANSFER

### Nutrient transformation

Once entering the environment, eDNAs is subjected to mixed environmental disturbances that determine their fate. Approximately 10% (w/w) of DNA molecules is composed of phosphorus, which makes it an important source of phosphorus in the natural environment. As reported by Turner et al. [109], approximately 22%–53% and 9%–13% of extractable phosphorus came from DNA in natural wetland soil and tundra soil, respectively. Similarly, eDNAs‐phosphorus accounted for 3% of the total phosphorus in marine sediment [110]; the proportion of phosphorus remineralization in eDNAs was 17% of the total phosphorus remineralization in the top 10‐cm sediment, indicating that eDNAs also significantly drove the phosphorus transformation in marine sediment [16]. Meanwhile, eDNAs degradation also provided about 4% carbon, 7% nitrogen, and 47% phosphorus for planktons [16].

Nuclease released by soil bacteria degraded eDNAs, and generated nutrient elements that was available for uptake of bacteria and plants [18, 111]. As reviewed by David et al. [12], parts of the elements can be reused for nucleic acid synthesis, while some are further degraded into PO_4_^3^^−^, CO_2_, and NH_3_. On one hand, exogenous eDNAs can be used as the sole carbon source of *E. coli*. In contrast to the wild‐type *E. coli*, the mutant bacteria could not survive due to its inability to use eDNAs. Through genome sequencing of both wild‐type and mutant bacteria, the *com* gene which harbored was found to be the gene that determined whether the bacterium had the capacity of using eDNAs or not [112]. *Haemophilis influenza* and *Nesseria gonorrhoeae* were both found to uptake homologous and heterologous eDNAs for nutrient elements and DNA damage reparation. However, it is important to note that only part of eDNAs can be degraded by nuclease and available for nutrient transformation in the environment [86].

### Gene exchange

**Figure 4:**
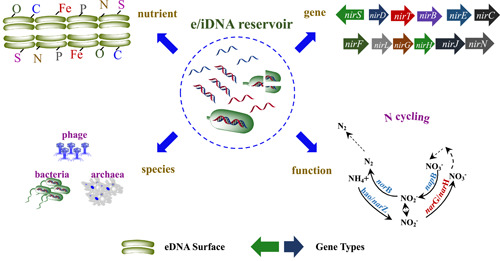
Biological function and gene communication for extracellular or intracellular DNA (eDNA/iDNA). Specifically, *norB*, *napB*, *hao*, and so forth are specific nitrogen gene types.

eDNAs usually persist in environments and were recognized as an archive of historical biological changes; iDNAs is the biological genetic information living at the moment [42, 113]. It has become a hot topic to distinguish eDNAs from iDNAs in environment, and determine the corresponding biological information in both DNA fraction [27, 47]. As figured by high throughput sequencing, both iDNAs and eDNAs were consisted of abundant eukaryotic and prokaryotic information [26, 43, 45, 114, 115]. For instance, Corinaldesi et al. [29] detected high abundant unicellular eukaryotes (10^9^–10^10^ copies g^−1^) and prokaryotes (10^9^–10^11^ copies g^−1^) in the sediment of the Black Sea despite high DNase activities. Considering that not all the eDNAs released can be restored in the environment, the biological composition shared similar information while showing differences between the two DNA fractions (Figure 4).

### Functional genes

Functional genes commonly include four groups of genes that present extensively in environments, which play critical roles in maintaining the ecological functioning of the host microorganisms. The first one is the biogeochemical cycling genes (BCGs) that regulate the biogeochemical cycling of nutrient elements, such as nitrifying gene *amoA* and denitrifying genes *nosZ*, *nirK*, *nirS*. The second group consists of antibiotic resistance genes (ARGs), including tetracycline resistance genes *tetW*, *tetM*, *tetQ*. The third one mainly includes phylogenetic marker genes (PMGs), including the GTP‐binding protein marker gene *lepA*, and peptide elongation factor G gene *fusA*. The last classification consists of plant pathogenicity genes (PPGs), the representative of which are thaxtomin biosynthetic/regulating genes *txt* gene cluster. In this study, we mainly focused on the first BCGs and the ARGs characteristics in eDNAs and iDNAs due to limited information about the other gene groups.

Metabarcoding and quantitative PCR techniques have been primarily applied to investigate the BCGs in eDNAs/iDNAs in the environment [116]. Maria et al. [47] selected carbon‐BCGs (*cell*, *xyl*, *alfagluc*, *betagluc*), phosphorus‐BCGs (*acP*, *alkP*, *bisP*, *leu*, *lys*), sulfur‐BCG (*aryS*) as primers of quantitative PCR to explore the activity of the corresponding enzymes in various forest soils. As expected, eDNAs and iDNAs both concluded abundant BCGs in various soils, but with varying genes and species information. However, it still remains unknown whether these BCGs in eDNAs and iDNAs can be transformed horizontally through the bacterial community, and further impact the matter cycling and energy flow [117].

### Antibiotic resistance genes

The abusive application of antibiotics for the therapeutic or preventing purpose has resulted in the large amount of residue of antibiotics, thereby inducing the corresponding resistance genes in various ecosystems, which can be further disseminated by mobile genetic elements, such as plasmids, transposons, and integrons [8, 36, 118–120]. ARGs in the environments can be classified as intracellular antibiotic resistance genes (iARGs) and extracellular antibiotic resistance genes (eARGs). iARGs can be transferred to progeny bacteria through self‐replication, or to other bacteria through conjugation and transduction; and eDNAs were integrated by recipient bacteria through natural transformation [8, 121]. Therefore, due to the persistence of eARGs and iARGs in the environment, it is worth investigating the risk of e/iARGs proliferation on public health and environmental security.

The presence of ARGs in both iDNAs and eDNAs have been detected in various environments, namely rivers, lakes, marshland, underground water, glaciers, reservoir, etc [8, 122–125]. For example, Zhang et al. [31] quantified the abundance of ARGs (*sul1*, *sul2*, *tetM*, *tetB*, *bla*_*TEM*_, *qnrS*) in the seawater and sediments collected in the Bohai Sea; the relative abundance of eARGs was 4.30 ± 1.30 × 10^−1^ and 2.60 ± 0.30 × 10^−3^ copies g^−1^ in seawater and sediment, respectively; and the level of eARGs was significantly higher than that of iARGs (*p* < 0.05). Similarly, the detection frequency and abundance of 22 ARGs in the Yangtze River estuary followed the order of biofilm> sediment> river water, the high concentration of antibiotics in the river environment stimulated the proliferation of ARGs in biofilms [122]. Meanwhile, more than 60% of ARGs detected in the biofilm and sediments were harbored by eDNAs, which was positively correlated with the content of total organic carbon in the environment. Combining with the result that the partition coefficiency of biofilm‐water was higher than that of sediment water, it could be concluded that biofilm is a critical source of ARGs in an aqueous system. Dong et al. [36] found the level of eARGs was in the range of 7.31 × 10^3^ and 1.16 × 10^10^ copies g^−1^ in medical wastewater, wastewater treatment plant, pig manure sludge, and municipal lake sediment, while the iARGs between 1.04 × 10^5^ and 2.74 × 10^12^ copies g^−1^; and the transformation frequency between eARGs and recipient *E. coli* DH 5α in adsorbed eARGs was clearly higher than that in free‐living eARGs in sediment. Compared to free‐living eDNAs, the adsorbed eDNAs was more prune to combine with recipient competent cell, the underlying mechanism still remains investigated.

## FUTURE PERSPECTIVES

Current research mostly concentrated on the environmental behavior of eDNAs and iDNAs, the role of eDNAs in biofilm formation, the abundance of eARGs and iARGs in various environments, and the correlation with corresponding host bacteria. However, it is of great significance to further care about the functional role of eDNAs and iDNAs in the environmental microbiome (e.g., soil, seawater, and marine sediments). To summarize, there are some aspects that deserve further attention in future study.

First, techniques that can precisely extract eDNAs and iDNAs separately are urgently expected as is the fundamental of the evaluation of the respective role of each DNA fraction play in the environment. The current method usually applied phosphate buffer to extract eDNAs and iDNAs, which extracting efficiency is unstable, and was impossible to distinguish free‐living, loosely, and tightly bound fractions. Consequently, it is difficult to evaluate the specific role of each DNA fraction play in the environment. With the development of the novel methodology, it is expected to contribute a proportion of dead or live bacteria, fungi, and so on to the existing eDNAs in the environment.

Meanwhile, from the macroscopic point of view, it is crucial to carry out a systematic investigation of the eDNAs/iDNAs in various environments. Given that most research was carried out in the interface between soil‐water, soil‐air, and water‐air system, a more broad scope study, that is, in situ soil‐seawater‐sediment, is essential for understanding the overall function of eDNAs/iDNAs and the ecological service they serve. Therefore, a global‐scale map of eDNAs/iDNAs distribution and environmental functions is expected to be drawn in near future.

Retrospectively, the functional genes harbored in eDNAs and the underlying mechanisms of gene transformation between eDNAs and recipient bacteria is another perspective which directs the development of the eDNAs' ecological service. For instance, employing stable isotope labeling and next‐generation sequencing techniques to compare the composition, abundance, and functions of the genes included in eDNAs/iDNAs, thereby understanding the role of both DNA fractions in nutrient cycling, historical dynamics in the microbial community, and varying niches occupied in environments.

Exploring the separated role of eDNAs and iDNAs in the environment, it contributes to a better understanding of the tactics of the microbial community to maintain their ecological functioning through active (iDNAs) and passive (eDNAs) pathways. Alongside with the current focus on the research of iDNAs, it is the proper time to bring eDNAs into sight, and investigate their contribution to the important matter cycling and genetic information transmission in both soil and marine environments. More specifically, more attention is needed to be paid to the involvement of eDNAs in biofilm formation and horizontal gene transfer facilitated by eDNAs through transformation to maintain the stability of microbial community, therein sustaining the ecological service in soil, seawater, and sediment environments.

## AUTHOR CONTRIBUTIONS

All authors contributed intellectually to and agreed to this submission. Mao Ye and Yu Shi contributed to conceptualization. Mao Ye, Zhongyun Zhang, and Mingming Sun contributed to the original draft writing, while Yu Shi contributed to the review and editing of the draft. All authors read and approved the final manuscript.

## CONFLICT OF INTEREST

The authors have declared no competing interests.

## Data Availability

No new data and script used in this study. Supplementary materials (figures, tables, scripts, graphical abstract, slides, videos, Chinese translated version and update materials) may be found in the online DOI or iMeta Science http://www.imeta.science/.
